# Management of Antithrombotic Therapy in Left Ventricular Thrombus: A Position Paper of the Italian Society of Hemostasis and Thrombosis (SISET)

**DOI:** 10.1055/a-2702-2298

**Published:** 2025-09-25

**Authors:** Emanuele Valeriani, Arianna Pannunzio, Danilo Menichelli, Domenico Prisco, Walter Ageno, Daniele Pastori, Pasquale Pignatelli

**Affiliations:** 1 Department of General Surgery, Surgical Specialty and Anesthesiology, Sapienza University of Rome, Rome, Italy; 2 Department of Infectious Disease, Azienda Ospedaliero-Universitaria Policlinico Umberto I, Roma, Italy; 3 Dipartimento di Medicina Sperimentale e Clinica, Università di Firenze, Florence, Italy; 4 Department of Medicine and Surgery, University of Insubria, Varese, Italy; 5 Department of Medical and Cardiovascular Sciences, Sapienza University of Rome, Rome, Italy; 6 IRCCS Neuromed, Pozzilli, Italy

**Keywords:** acenocoumarol, anticoagulants, heparin, venous thromboembolism, warfarin

## Abstract

Left ventricular thrombus (LVT) represents a potential life-threatening condition burdened by a significant risk of systemic embolism. Despite the relevance of the disease, there are scanty data on antithrombotic management of LVT mostly deriving from small observational studies and few randomized controlled trials. It has been reported that anticoagulant therapy reduces the rate of thrombus formation, allows thrombus resolution in most cases, and limits the risk of embolic complications. Several issues, however, still remain unresolved and clinicians caring for these patients have to decide on the need and on the regimen of antithrombotic therapy based on their expertise and data from different clinical scenario. This position paper of the Italian Society of Hemostasis and Thrombosis (SISET) aims to provide practical advice and guidance in the form of text, tables, and figures for clinicians dealing with LVT. Relevant clinical questions related to LVT have been identified concerning the identification of patients at risk; the role of anticoagulant prophylaxis on LVT development; the type, dose, and duration of anticoagulant therapy; and the management of patients receiving concomitant antiplatelet therapy. A systematic search has been performed to identify available evidence on the topic that has been carefully and critically reviewed by the national expert authors to support the suggestions and recommendations.

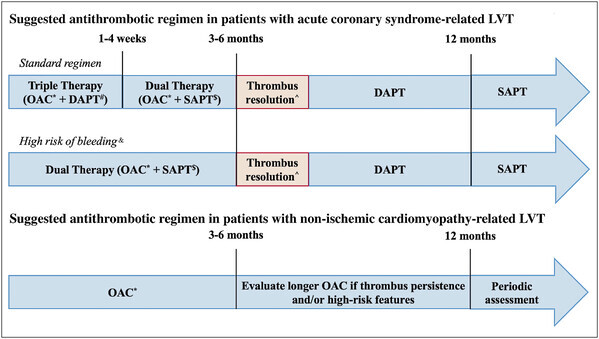

## Introduction

Left ventricular thrombus (LVT) represents a potential life-threatening condition due to the high risk of stroke or systemic embolism. The identification of predisposing factors and of patients at higher risk, and the decisions on prevention and treatment strategies for LVT, and on the optimal follow-up management remain challenging and not completely understood due to scanty information that mostly derives from observational studies and few small randomized controlled trials (RCTs). This position paper aims to provide clinicians with practical advice on how to manage antithrombotic drugs for the prevention and treatment of LVT.

## Consensus Document Proposal and Decision-making Process

This position paper has been developed according to the International Society on Thrombosis and Hemostasis (ISTH) Guidance Development Process and Manuscript Preparation. 1

Briefly, the proposing author submitted the proposal to the Methodological Board of the Italian Society of Hemostasis and Thrombosis (SISET) for the development of this position paper along with a list of collaborating expert authors. The SISET Methodological Board formally approved the proposal, and suggested the addition of other national expert authors selected based on their expertise in the field; one of the chairs of the Board also participated in the working group. After in-depth discussion of the draft, all the authors agreed on final recommendations.

## Consensus Document Focus and Target Population

This document is focused on the management of antithrombotic therapy in LVT. We acknowledge that the diagnostic management or the use of complementary therapies other than antithrombotic for patients at risk for or with LVT did not represent the focus of this position paper and was not systematically searched or specifically discussed in the text.

## Clinical Questions

All the members of the Panel were entrusted by the chairs to define clinical questions concerning the most relevant issues about the clinical management of antithrombotic therapy in patients with LVT ( Table 1 ). The Panel agreed on identifying five questions, listed below, as the most relevant for the purpose of this guidance. Such questions provided the framework to inform the systematic search of literature (systematic reviews and original studies).

**Table 1:** Questions and statements identified by panelists

***What are the epidemiology and risk factors for LVT?*** 1. An evaluation for LVT detection is recommended in patients with acute myocardial infarction and with high-risk features for LVT development (see Table 2 ). 2. An evaluation for LVT detection is suggested in patients with non-ischemic heart disease and high-risk features for LVT development (see Table 3 ).
***Is anticoagulation effective in preventing LVT?*** 1. Routine administration of anticoagulants to prevent LVT is not recommended. 2. Administration of anticoagulants for primary prophylaxis is suggested in patients with acute myocardial infarction and high-risk features for LVT development (see Table 2 ). 3. If primary prophylaxis with anticoagulants is prescribed, the administration of low-dose rivaroxaban (i.e., 2.5 mg q12h) is suggested for at least 1 month and up to 3 to 6 months after the acute myocardial infarction, on top of dual antithrombotic therapy and depending on bleeding risk. A careful evaluation of thrombotic and bleeding risks is recommended on a case-by-case basis to assess the benefits and risks of extending primary prophylaxis beyond the first month. 4. In patients with non-ischemic cardiomyopathy, a careful evaluation on a case-by-case basis is recommended to assess the need of a primary prophylaxis of LVT with anticoagulants.
***How to treat patients with LVT?*** 1. Oral anticoagulant therapy is recommended in patients with newly diagnosed LVT. 2. In case of VKAs administration, an INR range between 2.0 and 3.0—with concomitant therapeutic dose of heparin until the target INR is reached—as well as a TTR ≥70% is recommended to reach the highest possible rate of thrombus resolution and embolic events prevention. 3. The use of DOACs is mostly suggested in patients who are considered unsuitable and/or refusing VKAs.
***How long anticoagulant therapy should be continued in patients with LVT?*** 1. Anticoagulant therapy is recommended until LVT resolution and for a minimum of 3 to 6 months. 2. A longer duration of anticoagulant therapy—at least 12 months—is suggested in patients at high risk of thrombus persistence/recurrence and embolic complications (see Tables 2 and 3 ).
***Can anticoagulant therapy be safely administered along with antiplatelets?*** 1. In patients with LVT needing coronary reperfusion therapy, triple therapy with anticoagulants and dual antiplatelets is recommended for at least of 7 days and up to a maximum of 1 month, followed by dual therapy with anticoagulant and single antiplatelet. 2. At LVT resolution, resuming a second antiplatelet instead of anticoagulant therapy up to 12 months after the acute MI is suggested. A single antiplatelet is recommended lifelong after the first 12 months of therapy. 3. A different duration of concomitant anticoagulant and antiplatelet therapy is suggested on a case-by-case basis weighing bleeding and thrombotic risks.

Abbreviations: LVT, left ventricular thrombus; MI, myocardial infarction; VKAs, vitamin K antagonists.

## Literature Search

A comprehensive review of the literature of studies published from inception until June 2024 was performed on MEDLINE using the following terms: left ventricular thrombus, warfarin, acenocoumarol, apixaban, dabigatran, edoxaban, rivaroxaban, anticoagulants, low-molecular-weight heparin, unfractionated heparin. The search strategy is listed in Supplementary Table S1 (available in the online version only). A total of 92 abstracts were identified as relevant to the selected questions and were manually reviewed by the authors; a total of 65 articles were finally selected. From the list of references available in the selected articles 25 articles were identified.

## Guidance Statements

After reviewing the available literature and summarizing the current evidence, guidance statements were generated following conference-call discussions among the authors. All guidance statements were voted for agreement and revised based on comments. Strong consensus was defined as >90% agreement among the panelists, and moderate agreement defined as 75 to 89%, as previously described. 2 The wording “recommend” indicates a strong consensus among the panel members and/or the availability of high-quality evidence. The wording “suggest” reflects a weak guidance statement with moderate consensus among the panel members and/or the availability of lower-quality evidence.

## Questions and Statements

### What are the Epidemiology and Risk Factors for LVT?

Before the introduction of coronary reperfusion therapy for acute coronary syndrome, the incidence of LVT in patients with ST-elevation myocardial infarction was reported in a range from 20 to 40% and up to 60% in larger infarction of the anterior wall. 3 4 5 The incidence decreased after the introduction of early pharmacological and interventional coronary reperfusion therapy and nowadays the rate is reported to range between 4 and 15%. 6 7 The incidence of LVT reported in different Italian cohorts of patients with ischemic heart disease was similar, ranging from 5% in studies using trans-thoracic echocardiography to 19% in those using magnetic resonance. 8 9 This large difference in LVT incidence may be due to the different time lag from the onset of myocardial infarction and diagnostic evaluation, and the differences in included patients in terms of location and severity of myocardial infarction and type of LVT risk factors. 10 The diagnostic method may also affect incidence value. Cardiac magnetic resonance represents the gold standard for LVT diagnosis, while trans-thoracic echocardiography still remains the screening diagnostic tool in patients at risk for LVT development with its sensitivity improving thanks to the administration of contrast media. 11 However, magnetic resonance should be considered in the case of undiagnostic echocardiography and a high clinical suspicion of LVT. 12 Trans-esophageal echocardiography does not increase LVT assessment rate as it does not improve the visualization of the left ventricle apex. 11 Even if no sound data are available, contrast-enhanced coronary computed tomography showed great potential for LVT detection due to its very high spatial resolution (<1 mm), excellent evaluation of cardiac morphology, ease of use, and low costs. 13

LVT can be classified based on the time horizon since the onset of acute myocardial infarction as *“recent”* or *“non-recent”* if the diagnosis is made within 7 days or within 1 to 6 months after the acute myocardial infarction, respectively. Although LVT was recent in most of the cases, the non-recent form is quite rare (10% of patients) and may have different pathogenesis, depending on persistent systolic dysfunction of left ventricle. 14 15 16 17 LVT can also be categorized according to its morphological characteristics as *“mural”* if its borders are contiguous with the adjacent endocardium, or as *“protuberant”* if it protrudes into the left ventricle cavity. 14 Mural LVT is often undetected by trans-thoracic echocardiography because a close approximation of the thrombus to the adjacent akinetic wall can limit its detection. The risk of LVT is greater when the anterior wall—encompassing the perfusion territory of the left anterior descending coronary—or a large area of infarct is involved, and when there are a relevant delay in presentation to reperfusion time, a pre-angioplasty TIMI flow grade ≤1, and a reduced left ventricular ejection fraction (e.g., <40%) ( Table 2 ). 6 18 19 20 Nevertheless, LVT can be found in patients with smaller and inferior myocardial infarction, in patients with non-ST-elevation myocardial infarction as well as in patients with preserved or moderately reduced left ventricular ejection fraction. 5 21 22 The use of balloon angioplasty instead of drug-eluting or bare medical stents appeared to further increase the risk of LVT. 7 Other risk factors in ischemic heart disease patients include concomitant atrial fibrillation, left ventricle aneurysm, left heart valvular disease, acute or chronic deep venous thrombosis/pulmonary embolism, and alcohol abuse. 23 Also cardiac arrest is associated with an increased risk of LVT. 7 An inverse association with female sex has been also reported. 23

**Table 2:** Risk factors for LVT development in patients with acute myocardial infarction

Clinical conditions	High-risk features for LVT development
***Acute myocardial infarction***	Left ventricle antero-apical wall involvement
	ST-elevation acute myocardial infarction
	Large area of infarct
	Left-ventricle aneurysm
	Left heart valve disease
	Relevant delay in presentation to reperfusion time
	Pre-angioplasty TIMI flow grade ≤1

Abbreviation: LVT, left ventricular thrombus.

Patients with acute myocardial infarction-related LVT have an increased risk—up to 5-fold higher—of embolic complications compared with patients without LVT, whereas anticoagulated patients have a reduced risk of embolic complications compared with untreated ones. 24 A recent study including 155 patients with LVT diagnosis based on late gadolinium enhancement cardiovascular magnetic resonance imaging showed a 3.7% annualized rate of a composite of stroke, transient ischemic attack, and extracranial systemic embolism during a median follow-up of 3 years compared with the 0.8% of matched controls without LVT. 25 Most of embolic events, however, are acute ischemic stroke and occur within the first month after LVT occurrence with incidence values as high as 10%. 26 27 28 29 The embolic risk of LVT appeared to be higher in patients with protruding and mobile thrombus, in patients with LVT persistence and/or recurrence, and in patients not receiving anticoagulation. 24 26 30 31 32 33 An accurate clinical and radiological evaluation as well as a proper anticoagulant therapy is mandatory to ameliorate patients' prognosis. 34

Limited data are available about LVT in patients affected by non-ischemic heart diseases. Table 3 reports relevant causes and high-risk features for LVT development in patients with non-ischemic heart disease. Dilated cardiomyopathy represents the second most common underlying cause of LVT with incidence rates higher than 2%. 35 36 A reduced left ventricular ejection fraction, the presence of a scar, and a turbulent intracardiac flow are risk factors for LVT in dilated cardiomyopathy along with the presence of inflammation, hypercoagulability, and endocardial involvement of specific diseases (e.g., amyloidosis, eosinophilic myocarditis). 35 The incidence of LVT in Takotsubo syndrome was reported to be around 2%. 37 38 Incidence values were similar in European and Italian cohort of patients. 39 40 Patients with LVT and Takotsubo syndrome seem to have a higher prevalence of prior vascular disease (e.g., transient ischemic attack, stroke, myocardial infarction, peripheral artery disease, aortic plaques), a higher heart rate, and a lower left ventricular ejection fraction. 38 The interTAK Thrombus risk score—including apical involvement, prior vascular disease, left ventricular ejection fraction <30%, and white blood cells >10,000/mL—has been proposed to identify patients with Takotsubo syndrome at low (<3 points) or high risk (≥3 points) of LVT development. 38 An increased risk of thromboembolism in patients with left ventricle non-compaction also exists with history of stroke or transient ischemic attack and/or left ventricle dysfunction, being related to LVT formation in the deep intertrabecular recesses. 36 41 42 Several observational studies reported a relevant incidence of LVT (10–17%) in women with peripartum cardiomyopathy. 43 44 45 The use of specific intracardiac devices (e.g., edge-to-edge repair of mitral valve and left ventricular assisted devices) or anti-cancer treatments (e.g., anthracyclines) have been associated with an increased risk of LVT. 6 10 46 47 48 49 Finally, a small number of case reports and case series of LVT detection have been reported in patients with other forms of cardiomyopathy, including hypertrophic cardiomyopathy, cardiac amyloidosis, cardiomyopathy attributable to Chagas disease, and eosinophilic myocarditis. 50 51 52 53 54

**Table 3:** High-risk features for LVT development in patients with non-ischemic cardiomyopathy

Clinical conditions	High-risk features for LVT development
Chemotherapy-related cardiomyopathy 48	Left ventricular restrictive filling pattern and/or ejection fraction <30%
Takotsubo syndrome 37 38 39	Left ventricle dysfunction (ejection fraction <30%) and/or apical ballooning
Peripartum cardiomyopathy 44 45	Bromocriptine administration and/or ejection fraction <35%
Dilated cardiomyopathy 35 36	Blood stasis in dilated left ventricle, presence of a scar and of a turbulent intracardiac flow
Severe functional mitral regurgitation 46	Edge-to-edge Mitra Clip intervention
Left ventricular assisted devices 47	Device malfunction and embolism
Hypertrophic cardiomyopathy 50	Apical aneurysm
Left ventricular non-compaction 36 42	History of stroke or transient ischemic attack and/or left ventricle dysfunction
Cardiac amyloidosis 52	Amyloid light chain type and/or left ventricle restrictive filling pattern
Cardiomyopathy due to Chagas disease 53	Apical aneurysm
Eosinophilic myocarditis 54	Prior embolic episode

Abbreviation: LVT, left ventricular thrombus.

#### Statements

1. An evaluation for LVT detection is recommended in patients with acute myocardial infarction and with high-risk features for LVT development (see Table 2 ).
2. An evaluation for LVT detection is suggested in patients with non-ischemic heart disease and high-risk features for LVT development (see Table 3 ).

### Is Anticoagulation Effective in Preventing LVT?

Identification of patients in whom primary prophylaxis with anticoagulants may have a favorable risk–benefit profile is not well understood. 55

Even if limited by the heterogeneity of treatment regimens in terms of type, dose, and duration of anticoagulation and by the differences in diagnostic methods and treatments used in the pre-reperfusion era, a meta-analysis of four RCTs showed a reduced probability of LVT development in patients receiving anticoagulation compared with no treatment (odds ratio 0.32; 95% confidence interval, 0.20 to 0.52). 24 No data on bleeding events were reported. 24 In the same time period, 776 patients with acute anterior myocardial infarction were randomized in the FRAMI study to receive dalteparin (150 U/kg q12h for 9 ± 2 days) or placebo on top of thrombolytic therapy with intravenous streptokinase and aspirin. 56 Dalteparin significantly reduced LVT formation (absolute risk difference, 8.1%; number needed to treat, 12) at the cost of an increased rate of major bleeding (absolute risk difference, 2.6%; number needed to harm, 39). 56 No differences have been reported in terms of systemic embolism (1.3% in both groups). 56 The administration of dalteparin in such a short period surely limited the interpretation of results as a not negligible part of LVT develops after the first days from the acute myocardial infarction. More recently, a smaller RCT randomized 60 patients with acute anterior myocardial infarction to receive enoxaparin (1 mg/kg q12h for 1 month) or warfarin (INR between 2 and 3 for 3 months) on top of antiplatelet therapy. 57 Even if limited by different treatment durations, fewer patients in the warfarin group than in the enoxaparin group had an LVT at 3.5 months of follow-up (4% versus 15%, respectively). 57 At 1 and 3 months of follow-up, there were no stroke and systemic embolization in either group. 57 The incidence of any bleeding requiring treatment was similar (7% versus 4%). 57 In the last published RCT, a total of 279 patients with acute anterior myocardial infarction were randomized to receive low-dose rivaroxaban (2.5 mg q12h) or placebo on top of dual antithrombotic therapy for 1 month. 58 Patients in the rivaroxaban group had a significantly lower risk of LVT than controls (0.7% versus 8.6%; hazard ratio, 0.08 ; 95% confidence interval, 0.01 to 0.62) with a doubled and apparently not significant increase in the risk of bleeding events (3.6% versus 1.7%; hazard ratio, 2.08; 95% confidence interval, 0.38 to 11.33). 58 Furthermore, patients in the rivaroxaban group had a trend toward a reduced risk of systemic embolism compared with controls (0.7% versus 2.9%; hazard ratio, 0.51; 95% confidence interval, 0.09 to 2.69). 58 It should be acknowledged that this RCT included only patients from China, most of the included patients had a preserved or moderately reduced left ventricular ejection fraction, and that there was a high rate of drop-out (more than 15%) possibly limiting the generalizability and the robustness of results. 58

Few data were available on the primary prevention in patients with LVT of non-ischemic origin. Even though limited by the observational design, a recent observational study in patients with dilated cardiomyopathy showed similar incidence of LVT detection in patients receiving warfarin (2.9%) and lower incidence in patients receiving direct oral anticoagulants (DOACs) (1.3%) for different indications (e.g., atrial fibrillation) compared with untreated patients (2.7%). 35 In these latter cases, the administration of anticoagulants as primary prophylaxis must be carefully evaluated on a case-by-case basis taking into account specific patients' characteristics, thrombotic and bleeding risks, and preferences.

#### Statements

1. Routine administration of anticoagulants to prevent LVT is not recommended.
2. Administration of anticoagulants for primary prophylaxis is suggested in patients with acute myocardial infarction and high-risk features for LVT development (see Table 2 ).
3. If primary prophylaxis with anticoagulants is prescribed, the administration of low-dose rivaroxaban (i.e., 2.5 mg q12h) is suggested for at least 1 month and up to 3 to 6 months after the acute myocardial infarction, on top of dual antithrombotic therapy and depending on bleeding risk. A careful evaluation of thrombotic and bleeding risks is recommended on a case-by-case basis to assess the benefits and risks of extending primary prophylaxis beyond the first month.
4. In patients with non-ischemic cardiomyopathy, a careful evaluation on a case-by-case basis is recommended to assess the need of a primary prophylaxis of LVT with anticoagulants.

### How to Treat Patients with LVT?

Data on the efficacy and safety of heparin treatment are sparse. 59 Unfractionated or low-molecular-weight heparin administration within few days from LVT diagnosis was associated with an acceptable rate of thrombus resolution and with no embolic complications. 60 61 The role of vitamin K antagonists (VKAs) in LVT has been investigated in several observational and randomized studies. Even though limited by the small sample size (60 patients overall), 60% of patients randomized to VKAs (international normalized ratio [INR] range 1.6 to 2) had a complete thrombus resolution within the first 3 months of follow-up compared with 45% of patients randomized to aspirin (650 mg daily) and 10% of untreated patients. 62 No patients in the VKAs group developed an embolic event compared with 15% of untreated patients. 62 Recent observational studies confirmed the effectiveness of VKAs in terms of both thrombus resolution and embolic events prevention. 63 The most used INR range was between 2.0 and 3.0. 63 However, the time in therapeutic range (TTR)—calculated by Rosendaal interpolation method or % of INR in range—should be used for the therapeutic management of these patients as it reflects the quality of VKAs therapy. It has been reported from studies in other setting (i.e., atrial fibrillation) that patients with TTR <60% have higher thrombotic, bleeding, and mortality rates than those with higher TTR. 64 Reaching TTR values of at least 70% is therefore advisable. 64 A recent study showed that a TTR <50% (in patients with an INR target between 1.6 and 2.6) was associated with a higher incidence of ischemic events (19.0% versus 2.9%) and a lower rate of thrombus resolution (78% versus 91%). 65

DOACs use is increasing over the years. 63 To date, five RCTs have been published comparing standard dose of DOACs (three studies with apixaban and two studies with rivaroxaban) with warfarin for 3 to 6 months. 63 66 Although limited by the small sample size and methodological issues (e.g., open-label design), no significant differences in terms of efficacy have been found between DOACs and VKAs. 67 The use of DOACs was also evaluated in several observational studies that showed a high rate of thrombus resolution and a reduction of embolic events in patients with dilated cardiomyopathy-related LVT. 63 68 69 70 The largest and up-to-date available meta-analysis reported a trend toward high risk of thrombus persistence and a trend toward lower risk of embolic events in patients treated with DOACs compared with patients treated with VKAs. 63 71 Although awaiting sound data, the use of DOACs may be a reasonable alternative to VKAs to be considered in patients who are unsuitable (e.g., difficulties in achieving therapeutically stable INR, inability to undergo frequent blood sample collection for INR monitoring) and/or refusing VKAs.

Anticoagulant therapy profile in non-ischemic form of cardiomyopathy is limited to small case series and case reports and clinical decision must be addressed on a case-by-case basis. 72 Among others, the case of two children with LVT who received VKAs was reported, with an uneventful follow-up. 73

The role of early thrombolysis was evaluated in small case series including patients with high-risk features (e.g., mobile thrombus and reduced left ventricular ejection fraction). Even though thrombolysis appeared as an effective and safe procedure in these small case series further data are necessary to support its administration. 74 75 Finally, a tendency toward fewer embolic complications has been reported in patients treated with surgical LVT removal (8 patients) versus conventional therapy (42 patients). 76 Prophylactic anticoagulation with warfarin for 3 to 6 months after surgical LVT removal appeared to not reduce the incidence of LVT recurrence and other clinical outcomes compared with no treatment. 77

#### Statements

1. Oral anticoagulant therapy is recommended in patients with newly diagnosed LVT.
2. In case of VKA administration, an INR range between 2.0 and 3.0—with concomitant therapeutic dose of heparin until the target INR is reached—as well as a TTR ≥70% is recommended to reach the highest possible rate of thrombus resolution and embolic events prevention.
3. The use of DOACs is mostly suggested in patients who are considered unsuitable and/or refusing VKAs.

### How Long Should Anticoagulant Therapy be Continued in Patients with LVT?

Data from observational studies showed that LVT resolution generally occur within the first 6 months of therapy with oral anticoagulation. 78 79 80 Thus, anticoagulant treatment was administered up to 6 months after LVT diagnosis in most cases. 63 Some resolution may occur beyond the first 6 months of therapy; also some embolic complications may develop during the long-term follow-up. 68 80 81 82 83 84 85 Similarly, thrombus recurrence after resolution has been reported to develop in 10 to 20% of patients and appeared to be associated with a high embolic risk. 81 84 86 An anticoagulant treatment duration longer than 3 to 6 months—and beyond 12 months—is associated with a reduced risk of events development compared with a shorter regimen of therapy. 87 88

Some variables have been identified to guide treatment duration. Recent thrombi, as detected by clinical characteristics and specific echocardiographic measures (i.e., strain rate analysis), were associated with a higher rate of resolution than non-recent ones (94% versus 7%) after 6 months of phenprocoumon therapy. 89 However, warfarin treatment appeared to be more effective than no treatment in patients with a non-recent thrombus also, both in terms of thrombus resolution and embolic complications. 90 Limited data also suggest that protuberant thrombi may resolve earlier than mural ones, possibly reducing their long-term thromboembolic potential. 12 Smaller baseline thrombus size was associated with a greater likelihood of LVT regression (hazard ratio, 0.66; 95% confidence interval, 0.45 to 0.96). 87 Even though anticoagulation withholding may be considered after LVT resolution—especially in patients without high-risk features and/or high bleeding risk—it should be acknowledged that a prolonged anticoagulation over 3 months is associated with a reduced risk of major adverse cardiovascular events (hazard ratio, 0.42 ; 95% confidence interval, 0.20 to 0.88) and with a trend toward lower rate of embolic complications (hazard ratio 0.46; 95% confidence interval, 0.18 to 1.14). 87 Furthermore, a recent observational study reported the incidence of LVT resolution was higher at 12 months than at 3 and 6 months of anticoagulation. 91 Although no data are available on optimal anticoagulant therapy duration in patients with reversible ventricle dysfunction (e.g., Takotsubo syndrome), the cumulative incidence of LVT resolution progressively increased over a 6-month period in a cohort of patients with dilated cardiomyopathy. 69

Among risk factors for LVT persistence, the presence of a reduced left ventricular ejection fraction and a left ventricle apical aneurysm are the most relevant ones. 84 Conversely, a preserved or a moderately reduced left ventricular ejection fraction appeared not to be associated with an increased risk of thrombus persistence. 91 Regarding LVT recurrence, left ventricle aneurysm and anticoagulant treatment continuation were associated, respectively, with an increase and a reduction in the risk of this outcome in a retrospective cohort of 115 patients including both ischemic and non-ischemic cardiomyopathy-related LVT. 86 From a clinical point of view, advanced age is a relevant risk factor for a worst prognosis in patients with LVT, having a lower incidence of thrombus resolution and a higher incidence of systemic embolism compared with young patients. 91 92 Similarly, the presence of heart failure and chronic kidney disease appeared to be other relevant prognostic risk factors that have been associated with a reduced rate of thrombus resolution at 6 months and a higher rate of embolic complications. 93 94 95

Visual summary reports the suggested antithrombotic regimens in patients with acute coronary syndrome–related and with non-ischemic cardiomyopathy-related LVT.

#### Statements

1. Anticoagulant therapy is recommended until LVT resolution and for a minimum of 3 to 6 months.
2. A longer duration of anticoagulant therapy—at least 12 months—is suggested in patients at high risk of thrombus persistence/recurrence and embolic complications (see Tables 2 and 3 ).

### Can Anticoagulant Therapy be Safely Administered Along with Antiplatelets?

Overall, the incidence of major bleedings in patients with LVT receiving anticoagulation ranges between 2 and 3% and appears to be similar to the incidence reported in other clinical setting (e.g., atrial fibrillation with or without acute coronary syndrome). 63 96 97 Concomitant antiplatelet therapy may be needed in specific subgroups of patients, and this may increase the bleeding risk. 98 Short-term triple therapy—i.e., less than 1 month—with dual antiplatelets and warfarin appeared to confer low rates of major bleeding (1.1%) in 180 patients undergoing interventional procedure for acute myocardial infarction, half of whom had LVT as an indication for oral anticoagulation. 99 Data from small RCTs comparing DOACs with warfarin in patients with LVT showed a comparable safety profile during triple therapy. No patient receiving DOACs (apixaban) and 4.7% of those receiving warfarin developed a major bleeding during follow-up, all beyond the first month and after triple antithrombotic therapy was stopped. 100 101 Results were similar in a small observational study that indirectly compared triple therapy with rivaroxaban (0%) or warfarin (3.2%) for a median duration of 8.5 months in patients with acute myocardial infarction. 102 A larger study on 159 patients with confirmed LVT, of whom nearly 70% were receiving concomitant single or dual antiplatelets, showed a bleeding incidence as high as 13.2% during a follow-up of 632 days. 87 No further data were reported on specific subgroups of patients or on the timing of bleeding in relation to antithrombotic treatment. 87

Concerning the duration of concomitant triple antithrombotic therapy and type and dosage of concomitant antiplatelet therapy, data may be extrapolated from available RCTs on LVT management and from previous RCTs and guidelines recommendation regarding different clinical settings (e.g., atrial fibrillation in patients needing coronary reperfusion therapy). 98 103 Triple therapy with an oral anticoagulant (DOACs or warfarin) and dual antiplatelets (aspirin and a P2Y12 inhibitor, preferably clopidogrel) was administered for at least of 7 days and up to a maximum of 1 month and followed by dual therapy with oral anticoagulant and a P2Y12 inhibitor. 98 103 In very high bleeding risk patients—and in medically managed acute coronary syndrome—it may be useful to consider treating patients with anticoagulants and a single antiplatelet agent instead of triple therapy. 104 105 At LVT resolution, aspirin was resumed instead of anticoagulant therapy and dual antiplatelet was continued up to 12 months after the acute myocardial infarction. Thus, the P2Y12 inhibitor was continued indefinitely. A summary of these treatment strategies is reported in Visual Summary.

#### Statements

1. In patients with LVT needing coronary reperfusion therapy, triple therapy with anticoagulants and dual antiplatelets is recommended for at least of 7 days and up to a maximum of 1 month, followed by dual therapy with anticoagulant and single antiplatelet.
2. At LVT resolution, resuming a second antiplatelet instead of anticoagulant therapy up to 12 months after the acute myocardial infarction is suggested. A single antiplatelet is recommended lifelong after the first 12 months of therapy.
3. A different duration of concomitant anticoagulant and antiplatelet therapy is suggested on a case-by-case basis weighing bleeding and thrombotic risks.

## Conclusion

Given the lack of a solid evidence-based strategy to identify and manage patients with LVT, a thorough clinical evaluation of risk factors for LVT is needed.

Even though the optimal management has yet to be identified, this position paper provides useful therapeutic indications to manage patients with LVT in daily clinical practice.
