# Supplementary material for: Management of Antithrombotic Therapy in Left Ventricular Thrombus: A Position Paper of the Italian Society of Hemostasis and Thrombosis (SISET)
Source: Thromb Haemost. 2025 Sep 25;126(8):765–74. doi: 10.1055/a-2702-2298 (PMC13421306; doi:10.1055/a-2702-2298)
Supplement: Supplementary file 1 — Supplementary Material [file 10-1055-a-2702-2298_29249396.pdf]

Supplementary Table S1. MEDLINE search strategy

Ovid MEDLINE(R) and Epub Ahead of Print, In-Process, In-Data-Review & Other Non-Indexed Citations, Daily and Versions <1946 to September 05, 2024>		
**Items searched**		**Number of references**
1	((ventricle or ventricular) adj3 thromb*).mp.	2999
2	warfarin.mp. or exp Warfarin/	34856
3	acenocoumarol.mp. or exp Acenocoumarol/	1817
4	apixaban.mp.	6039
5	dabigatran.mp. or exp Dabigatran/	6929
6	edoxaban.mp.	2462
7	rivaroxaban.mp. or exp Rivaroxaban/	8888
8	exp Anticoagulants/	248733
9	(heparin adj3 (low or molecular or unfractionated)).mp	23368
10	2 or 3 or 4 or 5 or 6 or 7 or 8 or 9	264301
11	1 and 10	715
